# Supplementary material for: Phenotypic and Genetic Analyses of In Vitro Embryo Production Traits in Chinese Holstein Cattle
Source: Animals (Basel). 2023 Nov 16;13(22):3539. doi: 10.3390/ani13223539 (PMC10668646; doi:10.3390/ani13223539)
Supplement: Supplementary file 1 [file animals-13-03539-s001.zip › animals-2670113-supplementary.pdf]

Supplementary Materials

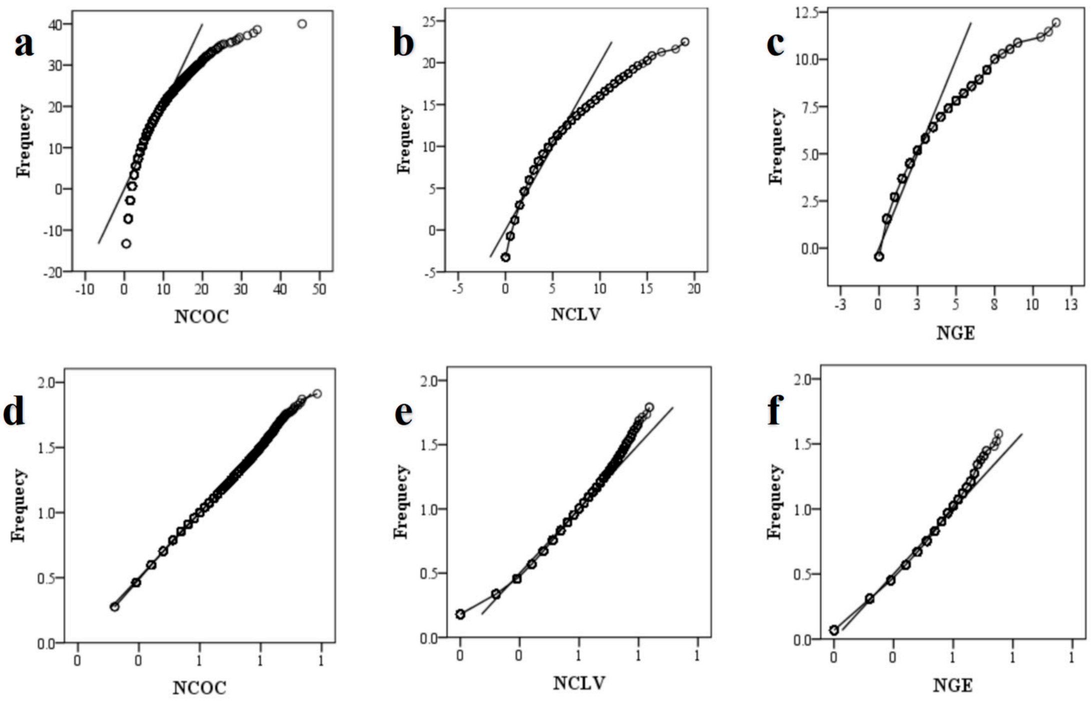

Supplemental Figure S1. Q_Q charts of three in-vitro embryo traits on observed scale (a-c) and on log-transformation scale (d-f)

NCOC = the number of cumulus-oocyte complexes; NCLV = the number of cleaved embryos; NGE = the number of grade I embryos

Supplemental Table S1. Genetic correlations for in-vitro embryo production traits on observed scale in Chinese Holstein cattle

Traits	NCLV	NGE	PCLV	PGE
NCOC	0.95±0.02	0.67±0.09	0.05±0.18	−0.14±0.17
NCLV		0.77±0.07	0.38±0.16	0.02±0.18
NGE			0.46±0.16	0.69±0.10
PCLV				0.42±0.17

NCOC = the number of cumulus-oocyte complexes; NCLV = the number of cleaved embryos; NGE = the number of grade I embryos; PCLV = the proportion of NCLV to NCOC; PGE = the proportion of NGE to NCOC.

Supplemental Table S2. Genetic correlations for in-vitro embryo production traits on log-transformed scale in Chinese Holstein cattle

Traits	NCLV	NGE	PCLV	PGE
NCOC	0.94±0.02	0.61±0.10	−0.02±0.18	−0.16±0.17
NCLV		0.69±0.09	0.36±0.16	−0.03±0.18
NGE			0.38±0.17	0.73±0.09
PCLV				0.42±0.17

NCOC = the number of cumulus-oocyte complexes; NCLV = the number of cleaved embryos; NGE = the number of grade I embryos; PCLV = the proportion of NCLV to NCOC; PGE = the proportion of NGE to NCOC.
